# Crystal structure of sodium di­hydrogen arsenate

**DOI:** 10.1107/S2056989017013470

**Published:** 2017-09-25

**Authors:** Joseph Ring, Lorenz Lindenthal, Matthias Weil, Berthold Stöger

**Affiliations:** a TU Wien, Getreidemarkt 9/164-SC, A-1060 Vienna, Austria; b Institute for Chemical Technologies and Analytics, Division of Structural Chemistry, TU Wien, Getreidemarkt 9/164-SC, A-1060 Vienna, Austria; c X-Ray Centre, TU Wien, Getreidemarkt 9, A-1060 Vienna, Austria

**Keywords:** crystal structure, isotypism, sodium arsenate, hydrogen bonding

## Abstract

Single crystals of the title compound, Na(H_2_AsO_4_), were obtained by partial neutralization of arsenic acid with sodium hydroxide in aqueous solution. The crystal structure of Na(H_2_AsO_4_) is isotypic with the phosphate analogue and the asymmetric unit consists of two sodium cations and two tetra­hedral H_2_AsO_4_^−^ anions. Each of the sodium cations is surrounded by six O atoms of five H_2_AsO_4_^−^ groups, defining distorted octa­hedral coordination spheres. In the extended structure, the sodium cations and di­hydrogen arsenate anions are arranged in the form of layers lying parallel to (010). Strong hydrogen bonds [range of O⋯O distances 2.500 (3)–2.643 (3) Å] between adjacent H_2_AsO_4_^−^ anions are observed within and perpendicular to the layers. The isotypic structure of Na(H_2_PO_4_) is comparatively discussed.

## Abstract 2

The crystal structure of Na(H_2_AsO_4_) is isotypic with that of Na(H_2_PO_4_). The two structures are compared with the aid of the *COMPSTRU* program.

## Chemical context   

Arsenic acid is triprotic and thus can form various salts, depending on the degree of deprotonation (H_2_AsO_4_^−^, HAsO_4_^2−^, AsO_4_^3−^), the condensation grade of the anion (mono-, di-, tri-, polyarsenate, *etc*) and the amount of water incorporated in the crystal. With respect to sodium arsenates, numerous crystal structures have been determined so far, including arsenic in tetra­hedral and/or in octa­hedral coordination by oxygen atoms. Arsenate structures with arsenic exclusively in tetra­hedral coordination resemble those of the related phosphates and in some cases show isotypism with them (marked by an asterisk): Na_3.25_(AsO_4_)(OH)_0.25_(H_2_O)_12_* (Tillmanns & Baur, 1971 ▸), Na_4_(AsO_4_)OH (zur Loye *et al.*, 2015 ▸), Na_2_(HAsO_4_)(H_2_O)_7_* (Baur & Khan, 1970 ▸; Ferraris *et al.*, 1971 ▸), Na(H_2_AsO_4_)(H_2_O) (Ferraris *et al.*, 1974 ▸), Na_3_(H_2_As_3_O_10_) (Driss & Jouini, 1990 ▸), Na_4_As_2_O_7_ (Leung & Calvo, 1973 ▸), Na(AsO_3_) (Liebau, 1956 ▸) and Na_5_(AsO_5_) (Haas & Jansen, 2001 ▸). Arsenate structures with arsenic in (complete or partial) octa­hedral coordination include Na(H_2_As_3_O_9_) (Driss, Jouini, Durif *et al.*, 1988 ▸), Na_3_(H_5_As_4_O_14_) (Driss & Jouini, 1989 ▸), Na(HAs_2_O_6_) (Dung & Tahar, 1978 ▸), Na_2_As_4_O_11_ (Driss, Jouini & Omezzine, 1988 ▸) and Na_7_As_11_O_31_ (Guesmi *et al.*, 2006 ▸). A detailed discussion of the structural principles and crystal chemical characteristics of arsenates with arsenic in octa­hedral coordination was given some time ago by Schwendtner & Kolitsch (2007 ▸).

Besides the Na:As 1:1 phase Na(H_2_AsO_4_)(H_2_O) another 1:1 phase, Na(H_2_AsO_4_), has been reported but without an additional water mol­ecule (Fehér & Morgenstern, 1937 ▸). To our surprise, a detailed structural investigation of this salt has not yet been reported. Therefore, we started crystal growth experiments and determined its structure and report here on the results.

## Structural commentary   

The crystal structure of Na(H_2_AsO_4_) is isotypic with that of Na(H_2_PO_4_) (Catti & Ferraris, 1974 ▸). The asymmetric unit of Na(H_2_AsO_4_) comprises two Na^+^ cations and two tetra­hedral AsO_2_(OH)_2_^−^ groups. The Na1^+^ cation shows a narrow Na—O bond-length distribution in the range 2.337 (2) to 2.498 (2) Å with a distorted octa­hedron as the corresponding coordination polyhedron. The bond-valence sum (Brown, 2002 ▸) for the Na1^+^ cation amounts to 1.15 valence units. The surrounding of the Na2^+^ cation is much more distorted, with a bond-length range from 2.338 (2) to 2.769 (3) Å under consideration of a sixfold coordination (bond-valence sum 0.92 valence units). There is an additional remote oxygen atom at a distance of 3.000 (3) Å from Na2^+^. Its contribution of 0.04 valence units to the bond-valence sum might be considered as too low for a significant inter­action, and therefore the first coordination sphere of Na2^+^ is discussed as that of a considerably distorted octa­hedron. The two di­hydrogen arsenate groups show the usual differences (Weil, 2000 ▸, 2016 ▸) between As—O and As—(OH) bonds, with two significantly shorter As—O bonds [mean 1.659 (8) Å] and two longer As—(OH) bonds [1.723 (12) Å].

**Figure 1:**
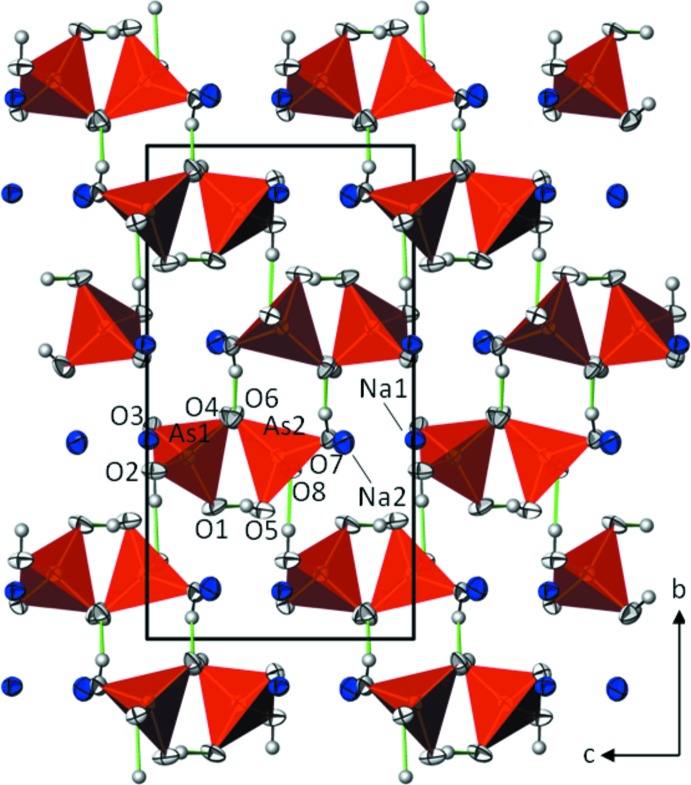
The crystal structure of Na(H_2_AsO_4_) in a projection along [100]. All atoms are depicted with displacement ellipsoids at the 97% probability level. Di­hydrogen arsenate tetra­hedra are given in polyhedral representation, Na^+^ cations as single ellipsoids without bonds to surrounding O atoms. H⋯O hydrogen bonds are illustrated with green lines.

In the crystal structure of Na(H_2_AsO_4_) the AsO_2_(OH)_2_ tetra­hedra are arranged in layers lying parallel to (010) with the Na^+^ cations approximately on the same level (Fig. 1 ▸). Strong, asymmetric hydrogen bonds [O⋯O distances between 2.500 (3) and 2.643 (3) Å, Table 1 ▸] between each of the OH groups of the two di­hydrogen arsenate tetra­hedra and O atoms of adjacent tetra­hedra significantly contribute to the crystal packing. These hydrogen bonds are both within a layer and towards adjacent layers (Fig. 1 ▸).

The differences between the isotypic arsenate and phosphate structures can mainly be seen in the *X*—O bond lengths of the anions (*X* = As, mean of 1.69 Å; *X* = P, mean of 1.55 Å), with Δ_max_(*X*—O) of 0.15 Å between arsenate and phosphate tetra­hedra. The difference with respect to the Na—O distances in the two structures is less pronounced, with Δ_max_(Na—O) = 0.10 Å. Relevant bond lengths of the isotypic crystal structures of Na(H_2_AsO_4_) and Na(H_2_PO_4_) (Catti & Ferraris, 1974 ▸) are compiled in Table 2 ▸. A more qu­anti­tative comparison of the two crystal structures with the help of the *COMPSTRU* routine (de la Flor *et al.*, 2016 ▸) revealed the following values: The degree of lattice distortion (*S*), *i.e*. the spontaneous strain (sum of the squared eigenvalues of the strain tensor divided by 3), is 0.0159; the maximum distance (*d*_max._), *i.e*. the maximal displacement between the atomic positions of paired atoms, is 0.1920 Å for atom pair O1; the arithmetic mean (*d*_av_) of the distances of all atom pairs is 0.1108 Å; the measure of similarity (Δ) (Bergerhoff *et al.*, 1999 ▸) is a function of the differences in atomic positions (weighted by the multiplicities of the sites) and the ratios of the corresponding lattice parameters of the structures and amounts to 0.049.

## Synthesis and crystallization   

The title compound was prepared following a procedure by Fehér & Morgenstern (1937 ▸). An arsenic acid solution (*ca* 65%_wt_) was partly neutralized with diluted NaOH solution using methyl red as indicator. The resulting solution was concentrated by heating. Standing of the solution overnight on a warm plate (*ca* 313 K) afforded colourless crystals with a lath-like form and maximal edge lengths of 0.5 mm.

## Refinement   

Crystal data, data collection and structure refinement details are summarized in Table 3 ▸. Starting coordinates and labelling of atoms were taken from the isotypic Na(H_2_PO_4_) structure (Catti & Ferraris, 1974 ▸). Hydrogen atoms were clearly discernible from difference maps and were refined with distance restraints *d*(O—H) = 0.85 (1) Å.

## Supplementary Material

Crystal structure: contains datablock(s) I, global. DOI: 10.1107/S2056989017013470/hb7706sup1.cif

Structure factors: contains datablock(s) I. DOI: 10.1107/S2056989017013470/hb7706Isup2.hkl

CCDC reference: 1575494

Additional supporting information:  crystallographic information; 3D view; checkCIF report

## Figures and Tables

**Table 1:** Hydrogen-bond geometry (Å, °)

*D*—H⋯*A*	*D*—H	H⋯*A*	*D*⋯*A*	*D*—H⋯*A*
O2—H1⋯O8^i^	0.85 (2)	1.75 (2)	2.595 (3)	175 (5)
O4—H2⋯O8	0.83 (2)	1.82 (2)	2.643 (3)	178 (4)
O5—H3⋯O1^ii^	0.85 (2)	1.73 (2)	2.566 (3)	171 (5)
O7—H4⋯O6^iii^	0.85 (2)	1.66 (2)	2.500 (3)	169 (4)

Symmetry codes: (i) 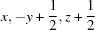; (ii) 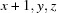; (iii) 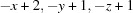.

**Table 2:** Comparison of bond lengths (Å) in the title compound and the isotypic phosphate analogue (Catti & Ferraris, 1974 ▸)

Bond	Na(H_2_AsO_4_)	Na(H_2_PO_4_)
Na1—O3^i^	2.337 (2)	2.355 (1)
Na1—O5^ii^	2.376 (2)	2.406 (1)
Na1—O3^iii^	2.382 (2)	2.371 (1)
Na1—O7	2.456 (2)	2.501 (1)
Na1—O2^iv^	2.459 (2)	2.436 (1)
Na1—O6^iv^	2.498 (2)	2.564 (1)
Na2—O8	2.338 (2)	2.334 (1)
Na2—O3^iv^	2.371 (2)	2.369 (1)
Na2—O1^ii^	2.419 (2)	2.433 (1)
Na2—O6^i^	2.586 (2)	2.601 (1)
Na2—O2^iv^	2.703 (3)	2.600 (1)
Na2—O4^i^	2.769 (3)	2.730 (1)
Na2—O7^v^	3.000 (3)	2.930 (1)
As/P1—O3	1.6484 (19)	1.499 (1)
As/P1—O1	1.657 (2)	1.508 (1)
As/P1—O4	1.730 (2)	1.592 (1)
As/P1—O2	1.736 (2)	1.597 (1)
As/P2—O6	1.663 (2)	1.523 (1)
As/P2—O8	1.668 (2)	1.519 (1)
As/P2—O7	1.711 (2)	1.562 (1)
As/P2—O5	1.713 (2)	1.572 (1)

Symmetry codes (i) −*x* + 1, −*y* + 1, −*z* + 1; (ii) *x*, −*y* + 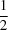, *z* − 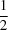; (iii) *x* + 1, *y*, *z* − 1; (iv) *x*, *y*, *z* − 1; (v) *x* − 1, *y*, *z*.

**Table 3:** Experimental details

Crystal data	
Chemical formula	Na(H_2_AsO_4_)
*M*_r_	163.93
Crystal system, space group	Monoclinic, *P*2_1_/*c*
Temperature (K)	100
*a*, *b*, *c* (Å)	7.0528 (14), 13.798 (3), 7.4792 (15)
β (°)	93.02 (3)
*V* (Å^3^)	726.8 (3)
*Z*	8
Radiation type	Mo *K*α
μ (mm^−1^)	9.32
Crystal size (mm)	0.12 × 0.08 × 0.01

Data collection	
Diffractometer	Bruker APEXII CCD
Absorption correction	Multi-scan (*SADABS*; Krause *et al.*, 2015 ▸)
*T*_min_, *T*_max_	0.534, 0.746
No. of measured, independent and observed [*I* > 2σ(*I*)] reflections	11092, 2651, 1890
*R*_int_	0.052
(sin θ/λ)_max_ (Å^−1^)	0.758

Refinement	
*R*[*F* ^2^ > 2σ(*F* ^2^)], *wR*(*F* ^2^), *S*	0.030, 0.059, 1.04
No. of reflections	2651
No. of parameters	125
No. of restraints	4
H-atom treatment	H atoms treated by a mixture of independent and constrained refinement
Δρ_max_, Δρ_min_ (e Å^−3^)	0.87, −0.86

Computer programs: *APEX3* and *SAINT* (Bruker, 2016 ▸), *SHELXL2016* (Sheldrick, 2015 ▸), *ATOMS* (Dowty, 2006 ▸) and *publCIF* (Westrip, 2010 ▸).

## supplementary crystallographic information

### Crystal data

**Table:**

Na(H_2_AsO_4_)	*F*(000) = 624
*M**_r_* = 163.93	*D*_x_ = 2.996 Mg m^−^^3^
Monoclinic, *P*2_1_/*c*	Mo *K*α radiation, λ = 0.71073 Å
*a* = 7.0528 (14) Å	Cell parameters from 2433 reflections
*b* = 13.798 (3) Å	θ = 3.1–31.8°
*c* = 7.4792 (15) Å	µ = 9.32 mm^−^^1^
β = 93.02 (3)°	*T* = 100 K
*V* = 726.8 (3) Å^3^	Lath, colourless
*Z* = 8	0.12 × 0.08 × 0.01 mm

### Data collection

**Table:**

Bruker APEXII CCD diffractometer	1890 reflections with *I* > 2σ(*I*)
ω and φ scans	*R*_int_ = 0.052
Absorption correction: multi-scan (*SADABS*; Krause *et al.*, 2015)	θ_max_ = 32.6°, θ_min_ = 2.9°
*T*_min_ = 0.534, *T*_max_ = 0.746	*h* = −10→10
11092 measured reflections	*k* = −20→20
2651 independent reflections	*l* = −11→11

### Refinement

**Table:**

Refinement on *F*^2^	Primary atom site location: isomorphous structure methods
Least-squares matrix: full	Hydrogen site location: difference Fourier map
*R*[*F*^2^ > 2σ(*F*^2^)] = 0.030	H atoms treated by a mixture of independent and constrained refinement
*wR*(*F*^2^) = 0.059	*w* = 1/[σ^2^(*F*_o_^2^) + (0.020*P*)^2^ + 0.0156*P*] where *P* = (*F*_o_^2^ + 2*F*_c_^2^)/3
*S* = 1.04	(Δ/σ)_max_ = 0.001
2651 reflections	Δρ_max_ = 0.87 e Å^−^^3^
125 parameters	Δρ_min_ = −0.86 e Å^−^^3^
4 restraints	

### Special details

**Table:**

Geometry. All esds (except the esd in the dihedral angle between two l.s. planes) are estimated using the full covariance matrix. The cell esds are taken into account individually in the estimation of esds in distances, angles and torsion angles; correlations between esds in cell parameters are only used when they are defined by crystal symmetry. An approximate (isotropic) treatment of cell esds is used for estimating esds involving l.s. planes.

### Fractional atomic coordinates and isotropic or equivalent isotropic displacement parameters (Å^2^)

**Table:**

	*x*	*y*	*z*	*U*_iso_*/*U*_eq_	
As1	0.32467 (4)	0.36827 (2)	0.84669 (4)	0.00611 (7)	
As2	0.82170 (4)	0.37010 (2)	0.50756 (4)	0.00595 (7)	
Na1	0.85834 (16)	0.40338 (8)	−0.00598 (15)	0.0091 (2)	
Na2	0.34819 (17)	0.39825 (9)	0.26282 (16)	0.0134 (3)	
O1	0.2417 (3)	0.26833 (14)	0.7478 (3)	0.0108 (4)	
O2	0.5315 (3)	0.34069 (15)	0.9726 (3)	0.0099 (4)	
O3	0.1910 (3)	0.42983 (14)	0.9809 (3)	0.0078 (4)	
O4	0.4015 (3)	0.44833 (15)	0.6878 (3)	0.0124 (4)	
O5	0.9202 (3)	0.26110 (14)	0.5709 (3)	0.0113 (4)	
O6	0.8540 (3)	0.45079 (14)	0.6715 (3)	0.0098 (4)	
O7	0.9303 (3)	0.40531 (15)	0.3188 (3)	0.0093 (4)	
O8	0.5931 (3)	0.34459 (14)	0.4613 (3)	0.0082 (4)	
H1	0.545 (7)	0.2797 (14)	0.970 (6)	0.055 (15)*	
H2	0.462 (5)	0.415 (3)	0.619 (5)	0.038 (13)*	
H3	1.026 (4)	0.270 (3)	0.627 (6)	0.071 (18)*	
H4	0.995 (5)	0.457 (2)	0.332 (6)	0.045 (13)*	

### Atomic displacement parameters (Å^2^)

**Table:**

	*U*^11^	*U*^22^	*U*^33^	*U*^12^	*U*^13^	*U*^23^
As1	0.00551 (13)	0.00631 (14)	0.00645 (13)	0.00067 (11)	−0.00029 (10)	−0.00017 (11)
As2	0.00609 (13)	0.00599 (14)	0.00564 (13)	−0.00119 (11)	−0.00081 (10)	0.00025 (11)
Na1	0.0087 (6)	0.0086 (5)	0.0099 (6)	0.0004 (4)	−0.0002 (4)	0.0003 (4)
Na2	0.0161 (6)	0.0132 (6)	0.0106 (6)	0.0027 (5)	−0.0038 (5)	−0.0001 (5)
O1	0.0101 (10)	0.0078 (10)	0.0141 (11)	−0.0005 (8)	−0.0031 (8)	−0.0037 (8)
O2	0.0077 (10)	0.0068 (9)	0.0147 (11)	0.0005 (8)	−0.0044 (8)	0.0006 (8)
O3	0.0062 (10)	0.0094 (9)	0.0078 (10)	0.0016 (7)	0.0007 (7)	−0.0011 (7)
O4	0.0150 (11)	0.0112 (10)	0.0114 (11)	0.0040 (9)	0.0059 (9)	0.0033 (8)
O5	0.0105 (10)	0.0064 (10)	0.0163 (11)	−0.0006 (8)	−0.0054 (9)	0.0012 (8)
O6	0.0101 (10)	0.0110 (10)	0.0083 (10)	−0.0033 (8)	0.0012 (8)	−0.0034 (8)
O7	0.0122 (10)	0.0091 (9)	0.0070 (9)	−0.0034 (9)	0.0038 (7)	−0.0008 (8)
O8	0.0058 (9)	0.0078 (9)	0.0108 (10)	−0.0011 (7)	−0.0013 (7)	0.0011 (8)

### Geometric parameters (Å, º)

**Table:**

As1—O3	1.6484 (19)	Na1—O6^iv^	2.498 (2)
As1—O1	1.657 (2)	Na2—O8	2.338 (2)
As1—O4	1.730 (2)	Na2—O3^iv^	2.371 (2)
As1—O2	1.736 (2)	Na2—O1^ii^	2.419 (2)
As2—O6	1.663 (2)	Na2—O6^i^	2.586 (2)
As2—O8	1.668 (2)	Na2—O2^iv^	2.703 (3)
As2—O7	1.711 (2)	Na2—O4^i^	2.769 (3)
As2—O5	1.713 (2)	Na2—O7^v^	3.000 (3)
Na1—O3^i^	2.337 (2)	O2—H1	0.847 (19)
Na1—O5^ii^	2.376 (2)	O4—H2	0.828 (18)
Na1—O3^iii^	2.382 (2)	O5—H3	0.847 (19)
Na1—O7	2.456 (2)	O7—H4	0.849 (19)
Na1—O2^iv^	2.459 (2)		
			
O3—As1—O1	120.05 (10)	O1^ii^—Na2—O4^i^	157.97 (8)
O3—As1—O4	107.40 (10)	O6^i^—Na2—O4^i^	73.32 (7)
O1—As1—O4	109.92 (11)	O2^iv^—Na2—O4^i^	90.18 (7)
O3—As1—O2	105.89 (10)	O8—Na2—O7^v^	128.32 (8)
O1—As1—O2	109.06 (10)	O3^iv^—Na2—O7^v^	72.63 (7)
O4—As1—O2	103.17 (10)	O1^ii^—Na2—O7^v^	74.48 (7)
O6—As2—O8	112.80 (10)	O6^i^—Na2—O7^v^	52.53 (6)
O6—As2—O7	111.60 (10)	O2^iv^—Na2—O7^v^	129.53 (8)
O8—As2—O7	111.00 (10)	O4^i^—Na2—O7^v^	125.50 (7)
O6—As2—O5	110.24 (10)	As1—O1—Na2^vi^	131.96 (12)
O8—As2—O5	104.21 (10)	As1—O2—Na1^vii^	135.72 (11)
O7—As2—O5	106.56 (10)	As1—O2—Na2^vii^	86.99 (8)
O3^i^—Na1—O5^ii^	161.36 (9)	Na1^vii^—O2—Na2^vii^	109.31 (9)
O3^i^—Na1—O3^iii^	90.20 (7)	As1—O2—H1	107 (3)
O5^ii^—Na1—O3^iii^	89.30 (8)	Na1^vii^—O2—H1	104 (3)
O3^i^—Na1—O7	86.19 (8)	Na2^vii^—O2—H1	111 (3)
O5^ii^—Na1—O7	75.23 (8)	As1—O3—Na1^i^	130.46 (11)
O3^iii^—Na1—O7	83.49 (8)	As1—O3—Na2^vii^	101.02 (9)
O3^i^—Na1—O2^iv^	102.04 (8)	Na1^i^—O3—Na2^vii^	100.03 (8)
O5^ii^—Na1—O2^iv^	80.75 (8)	As1—O3—Na1^viii^	122.85 (11)
O3^iii^—Na1—O2^iv^	166.71 (9)	Na1^i^—O3—Na1^viii^	89.80 (7)
O7—Na1—O2^iv^	102.26 (9)	Na2^vii^—O3—Na1^viii^	110.53 (9)
O3^i^—Na1—O6^iv^	79.94 (8)	As1—O4—Na2^i^	128.10 (12)
O5^ii^—Na1—O6^iv^	118.47 (8)	As1—O4—H2	105 (3)
O3^iii^—Na1—O6^iv^	83.21 (8)	Na2^i^—O4—H2	99 (3)
O7—Na1—O6^iv^	160.71 (8)	As2—O5—Na1^vi^	134.83 (12)
O2^iv^—Na1—O6^iv^	93.76 (8)	As2—O5—H3	111 (3)
O8—Na2—O3^iv^	156.69 (9)	Na1^vi^—O5—H3	113 (3)
O8—Na2—O1^ii^	86.89 (8)	As2—O6—Na1^vii^	122.07 (11)
O3^iv^—Na2—O1^ii^	90.22 (8)	As2—O6—Na2^i^	128.58 (11)
O8—Na2—O6^i^	122.07 (8)	Na1^vii^—O6—Na2^i^	90.36 (7)
O3^iv^—Na2—O6^i^	77.53 (7)	As2—O7—Na1	137.29 (12)
O1^ii^—Na2—O6^i^	126.96 (8)	As2—O7—Na2^ix^	126.26 (11)
O8—Na2—O2^iv^	92.76 (8)	Na1—O7—Na2^ix^	90.85 (7)
O3^iv^—Na2—O2^iv^	63.95 (7)	As2—O7—H4	114 (3)
O1^ii^—Na2—O2^iv^	81.03 (8)	Na1—O7—H4	102 (3)
O6^i^—Na2—O2^iv^	132.91 (8)	Na2^ix^—O7—H4	61 (3)
O8—Na2—O4^i^	73.31 (7)	As2—O8—Na2	137.60 (11)
O3^iv^—Na2—O4^i^	104.07 (8)		

Symmetry codes: (i) −*x*+1, −*y*+1, −*z*+1; (ii) *x*, −*y*+1/2, *z*−1/2; (iii) *x*+1, *y*, *z*−1; (iv) *x*, *y*, *z*−1; (v) *x*−1, *y*, *z*; (vi) *x*, −*y*+1/2, *z*+1/2; (vii) *x*, *y*, *z*+1; (viii) *x*−1, *y*, *z*+1; (ix) *x*+1, *y*, *z*.

### Hydrogen-bond geometry (Å, º)

**Table:**

*D*—H···*A*	*D*—H	H···*A*	*D*···*A*	*D*—H···*A*
O2—H1···O8^vi^	0.85 (2)	1.75 (2)	2.595 (3)	175 (5)
O4—H2···O8	0.83 (2)	1.82 (2)	2.643 (3)	178 (4)
O5—H3···O1^ix^	0.85 (2)	1.73 (2)	2.566 (3)	171 (5)
O7—H4···O6^x^	0.85 (2)	1.66 (2)	2.500 (3)	169 (4)

Symmetry codes: (vi) *x*, −*y*+1/2, *z*+1/2; (ix) *x*+1, *y*, *z*; (x) −*x*+2, −*y*+1, −*z*+1.
